# Rubella Associated with Hemophagocytic Syndrome. First Report in a Male and Review of the Literature

**DOI:** 10.4084/MJHID.2012.050

**Published:** 2012-08-09

**Authors:** M. Koubâa, Ch Marrakchi, I. Mâaloul, S. Makni, L. Berrajah, M. Elloumi, B. Hammami, D. Lahiani, T. Boudawara, M. Ben Jemâa

**Affiliations:** 1 Department of Infectious Diseases, Hedi Chaker Hospital, Sfax 3029, Tunisia; 2 Department of Pathology, Habib Bourguiba Hospital, Sfax, Tunisia; 3 Department of Microbiology, Habib Bourguiba Hospital, Sfax, Tunisia; 4 Department of Clinic Hematology, Hedi Chaker Hospital, Sfax, Tunisia

## Abstract

A 22-year-old man was admitted to our hospital because of fever, skin rash and epistaxis. Physical examination revealed fever (39.5°C), generalized purpura, lymphadenopathy and splenomegaly. Blood tests showed pancytopenia. Bone marrow aspiration and biopsy showed hemophagocytosis with no evidence of malignant cells. Anti rubella IgM antibody were positive and the IgG titers increased from 16 to 50 UI/mL in 3 days. Therefore, he was diagnosed to have rubella-associated hemophagocytic syndrome. We report herein the first case in a man and the sixth case of rubella-associated hemophagocytic syndrome in the literature by search in Pub Med till March 2012.

## Introduction

Haemophagocytic syndrome (HS) is caused by a dysregulation in natural killer T-cell function, resulting in activation and proliferation of lymphocytes or histiocytes with uncontrolled haemophagocytosis and cytokine overproduction.^1^ The syndrome is characterised by fever, hepatosplenomegaly, cytopenias, liver dysfunction, and hyperferritinaemia. HS can be either primary, with a genetic etiology, or secondary associated with malignancies, autoimmune diseases, or infections.^2^

Rubella or German measles is a viral infection typically characterized by rash, fever, and lymphadenopathy. The rash is usually an erythematous, discrete maculopapular exanthem that begins on the face and spreads caudally. It usually disappears within three days but may persist for eight days.

HS associated with rubella is uncommon. We report the first case of rubella virus-associated HS in a previously healthy 22-year-old man and we review all reported cases of rubella associated with HS in the literature by search in Pub Med till March 2012.

### Case Presentation

**Figure 1:**
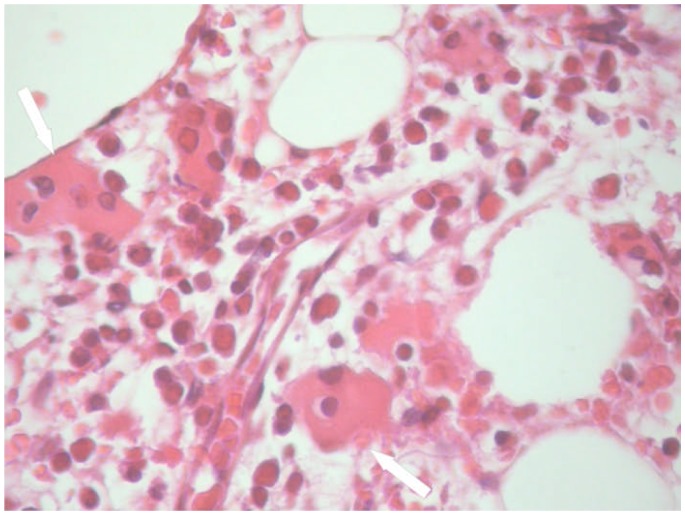
Hemophagocytosis in bone marrow biopsy (hematoxylin-eosin staining, ×400) of an 22-year-old man with rubella associated hemophagocytic syndrome.

A 22-year-old man was admitted to infectious diseases department with a 9-day history of fever, eruption, epistaxis and asthenia. Before admission, he had taken to a local hospital for fever and sore throat. He had treated with Cefpodoxime for 7 days. On admission, physical examination revealed fever (39.7 °C), generalized purpura and petechiae on the soft palate. There were several enlarged cervical, axillary and inguinal lymph nodes that were soft and tender. Abdominal examination revealed splenomegaly. Laboratory tests revealed the following: normocytic-normochromic anemia (hemoglobin 9.8 g/dL), white blood cell count 1700 cells/mm^3^ (neutrophils, 3%), platelet count 6 x10^9^/L. Results of the liver function tests were normal. Serum ferritin level was 6220 ng/mL and fibrinogen 132 mg/dl. Bone marrow aspiration and biopsy (Figure 1) revealed hemophagocytosis with no evidence of malignant cells. Anti rubella IgM antibody were positive and the IgG titers increased from 16 to 50 UI/mL in 3 days. The rubella serology was sought following the clinical and epidemiological context. Tests for common bacterial, mycobacterial, viral, fungal, auto-immune and tumoral causes of HS were negative. Therefore, he was diagnosed to have rubella-associated HS. The patient was treated by supportive measures including platelet transfusion. On discharge, physical examination was normal and her white blood cell count was 3500/mm^3^ (neutrophils, 42%), The hemoglobin level 10.8 g/dL and platelet count 159 x 10^9^/L.

## Discussion

We found in the literature five reports of rubella-associated HS in women.^3^–^7^ (Table 1) But, to our knowledge this is the first report in a man.

HS can be divided into primary or genetic HS and secondary or reactive HS. Primary HS usually occurs early in life and is associated with a higher mortality rate, while secondary HS occurs later in life and generally carries a better prognosis.^8^ Diagnosis of HS relies on specific clinical, laboratory, and histopathological findings proposed by the Histiocyte Society in 1991 and updated in 2004.^9^,^10^ The prognosis of infection-associated HS is better than other types of secondary HS. Risdall et al. introduced the term “virus-associated hemophagocytic syndrome” and set down criteria for its separation from malignant histiocytosis.^11^,^12^ The bone marrow hypoplasia may be due to the direct effect of viruses on the hemopoietic cells. The most common agents causing this syndrome are viruses, predominantly the herpes group, including Epstein-Barr virus, Herpes simplex, and Cytomegalovirus.^2^ Acquired HS is mostly associated with underlying diseases such as immunodeficiency, hematologic neoplasia, or autoimmune disease. Infection-associated HS is most common in immunocompromised patients such as renal transplant or lymphoma.^11^ The occurrence of HS in apparently immunocompetent patients may be explained by the basis of transient immunoparesis. There is no specific treatment for rubella. The anti-rubella vaccination represent the only way to prevent serious complications thus it should be indicated either in boys. Supportive therapy to address nutritional status, concomitant anemia, hemorrhagic complications, and secondary infections is therefore essential to optimize treatment outcomes.

## Conclusion

Rubella is a benign disease. HS may be observed in rubella, if bone marrow aspiration is performed on patients with cytopenia. Treatment consists on supportive care. No specific therapy for rubella infection is available. The evolution is generally favorable.

## Figures and Tables

**Table 1:** Summary of all reported patients with rubella associated hemophagocytosis syndrome in the literature

References	Marusawa 1994 [3]	Shinji 1996 [4]	Takenaka 1998 [5]	Takeoka 2001 [6]	Baykan 2005 [7]	Our case 2010
Age (Years)	57	4	29	26	2.5 months	22
Sex	F	F	F	F	F	M
Country	Japan	Japan	Japan	Japan	Turkey	Tunisia
Fever	+	+	+	+	+	+
Skin rash	+	+	+	+	+	+
Hepatosplenomegaly	NL	Splenomegaly	−	−	+	Splenomegaly
Lymph-adenopathy	NL	+	+	−	NL	+
Pancytopenia	+	+	bicytopenia	+	+	bicytopenia
Triglycerides > 265 mg/dL	NL	+	NL	NL	+	−
Fbrinogen < 1.5 g/dL	NL	+	NL	+	NL	+
Ferritin > 500/mg/dL	NL	+	−	+	NL	+
Hemophagocytosis	BMA	BMA	BMA	BMA	Liver necropsy	BMA and Bone marrow biopsy
Elevated transaminase	+	+	+	+	+	−
Immunocompetent host	+	+	+	− Idiopathic thrombocytopenic purpura treated with CS	+	+
Consultation deadline (Days)	NL	NL	2	4	NL	9
Diagnosis of rubella made by	Serology	Serology/EBV- IgM(+)	Skin biopsy/Serology	Serology/Serology for varicella- zoster virus(+)	Serology	Serology
Therapy	CS/IVIG	CS/IVIG	Antibiotics/IVIG/Platelet transfusion	IVIG/CS	Antibiotics/FFP/ES	Antibiotics/Platelet transfusion
Outcome	Alive	Alive	Alive (5 Y)	Alive (6 months)	Died	Alive (5 months)

M =Male; F = female; NL = not listed ; BMA= Bone marrow aspiration; CS = corticosteroids; IVIG = intravenous immunoglobin; Y= years; FFP= fresh-frozen plasma; ES= erythrocyte suspensions
